# Path to posttraumatic growth: The role of centrality of event, deliberate and intrusive rumination, and self blame in women victims and survivors of intimate partner violence

**DOI:** 10.3389/fpsyg.2022.1018569

**Published:** 2022-10-28

**Authors:** Aistė Bakaitytė, Alicia Puente-Martínez, Silvia Ubilos-Landa, Rita Žukauskienė

**Affiliations:** ^1^Institute of Psychology, Mykolas Romeris University, Vilnius, Lithuania; ^2^Department of Social Psychology and Anthropology, Faculty of Social Sciences, University of Salamanca, Salamanca, Spain; ^3^Department of Social Psychology, Faculty of Health Science, University of Burgos, Burgos, Spain

**Keywords:** posttraumatic growth, intimate partner violence, centrality of event, rumination, coping, self-blame

## Abstract

Increased interest in positive changes in the aftermath of traumatic events led researchers to examine assumptions about the process of posttraumatic growth (PTG). However, existing studies often use samples from mixed trauma survivors and investigate separate factors and their associations with growth. Therefore, the purpose of the current study was to examine the path from centrality of event to PTG involving intrusive and deliberate rumination and self-blame as a coping strategy in women survivors of intimate partner violence (IPV). The study sample consisted of 200 women with a history of IPV (ages 18–69, *M* = 44.79, *SD* = 12.94). Results of the path analysis indicated that higher centrality of event was related to higher levels of intrusive rumination which was positively related to self-blame and deliberate rumination eventually leading to PTG. Indirect effects from centrality of event to PTG through intrusive and deliberate rumination, and from intrusive to deliberate rumination through self-blame were examined. This study gave support to some theoretical assumptions of the process of PTG and pointed out problematic areas of investigation of coping strategies in this process.

## Introduction

Intimate partner violence (IPV) is defined as physical, sexual, and/or psychological harm caused by a current or former partner (World Health Organization, 2012). IPV is gender-based violence as most often women are the victims of their partners’ or ex-partners’ violence (World Health Organization, 2012) and they suffer more severe consequences of IPV compared to men (Ansara and Hindin, 2011). The dynamic of IPV is unique where the harm is done by a loved one, trusted partner, making this experience highly traumatic. Also, IPV involves controlling behaviors that isolate the victim from the outside world, and constant humiliation and mockery create an environment in which the victim loses the sense of self and her identity (Matheson et al., 2015). This kind of trauma causes well-known negative consequences such as PTSD, depression, and anxiety (Doane, 2010; Lilly et al., 2015; Chandan et al., 2020), but it can also lead to positive changes (Cobb et al., 2006; Valdez and Lilly, 2015).

For more than the past two decades, attention is given not only to negative consequences but also to positive changes experienced after traumatic events. The most widely used conceptualization of these changes was coined by Tedeschi and Calhoun (1995) and called posttraumatic growth (PTG). PTG refers to positive psychological changes that occur following the struggle with traumatic experiences (Tedeschi and Calhoun, 1995). The process of PTG is described in the model of PTG and the most recent version was published in 2018 (Tedeschi et al., 2018). The model indicates that traumatic experiences that challenge person’s core assumptions about the world and people in it are the ones that can initiate the PTG process. Changes can be experienced in the view of the self, relationships with others, and/or worldview and philosophy of life (Tedeschi and Calhoun, 1995; Tedeschi et al., 2018). Studies investigate PTG in various trauma survivors such as war veterans (Maguen et al., 2011), terminal illnesses (Chi et al., 2022), natural disasters (Jia et al., 2017), and accident survivors (Nishi et al., 2010), however, some traumatic experiences such as IPV still receive less attention (Elderton et al., 2017). The dynamics of IPV make it hard to apply the knowledge about PTG from other traumatic contexts. For this reason, it is important to investigate PTG in women victims and survivors of IPV, anticipating that the PTG process in this context may be different compared to other traumas.

In the model of PTG, authors use a broader understanding of traumatic experience than described in DSM-V indicating that not the event itself but the personal perception of it makes the experience traumatic (Tedeschi et al., 2018). This perception can be expressed through the centrality of event which refers to the degree to which an event becomes a central part of a person’s life story and identity (Berntsen and Rubin, 2006). If the traumatic event is perceived as central, it indicates that the experience became a turning point in which life is seen as one “before” and the other “after” (Tedeschi and Calhoun, 1995). This perception is an important precursor of PTG (Tedeschi and Calhoun, 1995), and studies with IPV and other trauma survivors confirm that the centrality of event is positively related to PTG (Groleau et al., 2013; Bakaitytė et al., 2021), indicating unambiguous importance of centrality of event in the process of PTG.

Another important factor for PTG is rumination. In general, rumination refers to repetitive thinking about something or “a cognitive “chewing the cud” (Cann et al., 2011, p. 138). Rumination belongs to the cognitive processing of trauma which is an essential part of the process of PTG (Tedeschi et al., 2018). Authors indicate two types of rumination: intrusive which is automatic and more present at the beginning of the process, and deliberate which is more reflective, appearing later in the process of PTG (Cann et al., 2011). Studies repeatedly indicate a positive relation between deliberate rumination and PTG (Ogińska-Bulik, 2016; Lafarge et al., 2020; Freedle and Kashubeck-West, 2021), and a negative or no relation between intrusive rumination and PTG (Stockton et al., 2011; Lafarge et al., 2020; Freedle and Kashubeck-West, 2021). The centrality of event is also found to be related to both types of rumination (Brooks et al., 2017; Kramer et al., 2020). Events that are central to a person initiate the cognitive processing of the traumatic experience, at first, through intrusive rumination, which is an inevitable part of trauma processing, but eventually, it must be transformed into more deliberate rumination to lead to PTG (Tedeschi et al., 2018). Besides theoretical assumptions, there is little empirical knowledge of what contributes to the shift from intrusive to deliberate rumination.

The model of PTG indicates that coping mechanisms also play a significant role in the cognitive processing of traumatic experiences. Coping is the response to stress that comes after a cognitive evaluation of the threat and possible responses to it (Lazarus and Folkman, 1984). It is assumed that in the process of PTG coping strategies help to manage distress caused by trauma and allow one to engage in cognitive processing through rumination (Joseph and Linley, 2005). Studies on coping and PTG often use so-called the fallacy of uniform efficacy (Bonanno and Burton, 2013) which is the tendency to combine different strategies into subtypes of coping based on different theoretical frameworks (e.g., adaptive/maladaptive, approach/avoidance, problem/emotion-focused coping). According to Bonanno and Burton (2013) concept of regulatory flexibility, coping is sensitive to the context of the event and environmental demands, meaning that the same strategy might be useful in one situation but not necessarily in others. This way of coping investigation is problematic as it gives generalized conclusions that most often could be debatable or questionable. This suggests that it is more useful to investigate separate coping strategies that are relevant to a given context rather than combining strategies in uniform constructs.

One of the forms of coping repeatedly reported in victims and survivors of IPV is self-blame (Reich et al., 2015; Ulloa et al., 2016). Although it partly comes from stigmatization in society (Kennedy and Prock, 2018) and is associated with negative consequences (Reich et al., 2015), it is also defined as a coping mechanism and can lead to positive outcomes (Tedeschi and Calhoun, 1995). Paradoxically, self-blame as a coping mechanism attributes control to oneself in this way helping to cope with what happened (Janoff-Bulman, 1979). Tedeschi and Calhoun (1995) indicate that self-blame helps to maintain beliefs that one has control in life and that positive changes are possible. In the context of IPV, the internal state of feeling responsible (self-blame) for experienced violence possibly stimulates thinking (rumination) about IPV experience and changes that are required to prevent it in the future. Therefore, considering that self-blame is very common in victims and survivors of IPV and that there are arguments in the literature suggesting its’ associations with rumination, this study focused on the analysis of the role of self-blame in the PTG process.

In categorizations of coping mechanisms, self-blame is most often attributed to avoidance coping. Studies using this conceptualization indicated a positive relation between PTG and avoidance coping in rehabilitation patients (Kunz et al., 2018), and 9–1–1 communicators who experienced childhood trauma (London et al., 2020), but in interpersonal violence survivors, avoidance coping was not related to PTG (Brooks et al., 2019). Little research investigates the relation between self-blame as an independent coping strategy and PTG, with exception of Frazier et al. (2004) who found no relation between behavioral self-blame and PTG in sexual assault victims. However, as argued before, it is possible that self-blame is more related to rumination leading to PTG than directly to the PTG itself.

The relationships between coping strategies and types of rumination are rarely investigated because most studies use them as direct predictors of PTG. Most extensive findings were reported by Cann et al. (2011) investigating relations between different coping strategies and types of rumination. Results indicated that only intrusive rumination predicted venting and mental disengagement (coping strategies) while deliberate rumination was not a significant predictor. Unfortunately, they did not include self-blame as a coping strategy but typically venting and mental disengagement are assigned to the same avoidance coping category as self-blame. Chi et al. (2022) found that deliberate rumination was positively associated with avoidance coping in people with HIV. Existing studies indicate mixed findings about relations between types of rumination and coping strategies, giving more attention to deliberate than intrusive rumination. Also, we could not find any study investigating relations between self-blame and types of rumination. However, Kamijo and Yukawa (2018) argue that feelings of regret and guilt motivate one to find meaning and can contribute to deliberate rumination. Moreover, the model of PTG (Tedeschi et al., 2018) indicates that coping helps the transition from intrusive to deliberate rumination. Therefore, it can be assumed that in the context of IPV self-blame plays a role in this transition.

Some studies indicate that the time since the traumatic event is an important factor contributing to PTG (Doane, 2010; Ulloa et al., 2016; Morgan and Desmarais, 2017). However, Prati and Pietrantoni (2009) conducted meta-analysis and did not find significant effect of time to PTG. Tedeschi et al. (2018) argue that for different people paths po PTG might differ and as some people may experience positive changes very early after the trauma, for other it may take years. Studies with victims and survivors of IPV support this by indicating that some positive changes can be experienced while still being in violent relationships (Young, 2007), but Cobb et al. (2006) highlight that the most significant PTG can be experienced after ending the violence. Results of the longitudinal investigation of PTG in IPV survivors showed that PTG increased for women who experienced IPV less than 2 years ago, and for those who experienced IPV more than 2 years ago PTG tend to be stable at relatively higher levels (Bakaitytė et al., 2022). These studies indicate that the time since the violence can be an important factor contributing to PTG in victims and survivors of IPV.

Although numerous studies are investigating PTG and confirming some aspects of the theory (e.g., Ulloa et al., 2016; Kramer et al., 2020; Lafarge et al., 2020), researchers are often concentrated on separate parts of the model and conduct studies with different or mixed trauma survivors (e.g., Cann et al., 2011; Lee et al., 2020). However, distinct types of trauma can have diverse impacts on PTG (Zoellner and Maercker, 2006; Lowe et al., 2020). For example, some authors argue that interpersonal traumas are more damaging to a core belief system than traumas by natural causes or accidents, therefore, potentially leading to more PTG (Ulloa et al., 2016). Others indicate that as interpersonal traumas are caused by others it is more difficult for survivors to make sense and meaning of them and this hinders growth (Meyerson et al., 2011). Moreover, coping can also differ by type of trauma (Bonanno and Burton, 2013) and this can also have an impact on the process of PTG. All these arguments highlight the importance to investigate PTG in homogenous types of trauma (Platte et al., 2022) or specific traumas such as IPV. This kind of investigation can give more focused and context-sensitive insights into the process of PTG.

Considering the need to investigate PTG in specific types of trauma and test theoretical assumptions of the cognitive processing part of the model of PTG as a whole, the purpose of the current study was to test the theoretical pathway from the centrality of event to PTG including rumination (intrusive and deliberate) and self-blame in women victims and survivors of IPV. We hypothesized that: (1) centrality of event will be positively associated with intrusive rumination; (2) intrusive rumination will be positively related to deliberate rumination; (3) self-blame will mediate the relation between intrusive and deliberate rumination; (4) intrusive and deliberate rumination will mediate the relation between centrality of event and PTG; (5) deliberate rumination will be positively associated with PTG.

## Materials and methods

### Participants

This study was a part of a larger research project on PTG of women victims and survivors of IPV in Lithuania. Thirty-seven experienced interviewers (only women) collected data from different regions of Lithuania. Interviewers went to the homes of potential study participants using the snowball method and information from the local social services. To identify victims and survivors IPV, questions about different forms of abuse were administered first. A participant was considered a victim or survivor of IPV if indicated at least one physical or sexual, or at least three psychological or economic violence incidents from their current or former partner. Stricter inclusion criteria for psychological and economic violence were selected considering the more nuanced nature of these types of abuse and some items possibly reflecting one-time conflicts occurring in the family (e.g., “Ignored, did not speak, did not answer questions,” “Demanded to tell me how and where I spend my money”). Participants completed self-reported questionnaires on paper at their homes if indicated that they feel safe doing so. The study was approved by the Ethics committee at Mykolas Romeris University.

The total sample consisted of 200 Lithuanian women (ages 18–69, *M* = 44.79, SD = 12.94) with a history of IPV. Almost two-thirds of participants had higher education (professional, college, or university degree), and 77.5% were employed. At the time of the study, 36.5% of participants were living with a partner, 33% were single, 19.5% had a partner but were not living together, 10.5% engaged in episodic relationships, and .5% did not indicate their relationship status. The IPV-related sample characteristics are presented in Table 1.

**Table 1:** IPV-related characteristics.

	*n* (%)
Forms of IPV in the sample	
Psychological violence	200 (100.0)
Economical violence	168 (84.0)
Physical violence	175 (87.5)
Sexual violence	130 (65.0)
Perpetrator(s)	
Current partner	54 (27.0)
Divorcing partner	34 (17.0)
One ex-partner	100 (50.0)
Multiple ex-partners	15 (7.5)
Time since the last violence incident	
Less than a week	9 (4.5)
More than a week	12 (6.0)
More than a month	27 (13.5)
More than a half year	28 (14.0)
More than a year	15 (7.5)
More than 2 years	30 (15.0)
More than 5 years	37 (18.5)
More than 10 years	22 (11.0)
More than 20 years	20 (10.0)
Received psychological help	
Yes	37 (18.5)
No	152 (76.0)
No response	11 (5.5)

Participants of the study could indicate more than one type of experienced IPV and more than one type of perpetrator(s).

### Measures

Posttraumatic growth was measured with the Short Form of Posttraumatic Growth Inventory (PTGI-SF; Cann et al., 2010) which consists of 10 items (e.g., “I changed my priorities about what is important in life”). Participants rated each item on a 6-point Likert-type scale ranging from 0 (I did not experience this change) to 5 (I experienced this change to a very great degree). The Cronbach’s alpha of the scale was .95.

Centrality of event was measured with the Centrality of Events Scale (CES; Berntsen and Rubin, 2006) which consists of seven items (e.g., “This event was a turning point in my life”). Participants rated each item on a 5-point Likert-type scale ranging from 1 (Totally disagree) to 5 (Totally agree). The Cronbach’s alpha of the scale was .89.

Intrusive and deliberate ruminations were measured with Event Related Rumination Inventory (ERRI; Cann et al., 2011). The measure consists of two subscales (10 items each) corresponding to intrusive (e.g., “I thought about the event when I did not mean to”) and deliberate (e.g., “I thought about whether I could find meaning from my experience”) rumination. For this study, we used five items for each scale. According to reported factor loadings of the scales (see Cann et al., 2011), all items were similar. Thus, we selected items that best fitted the sample and were not similar to other used measures (as this study was part of a larger study). Participants rated each item on a 4-point Likert-type scale ranging from 0 (Not at all) to 3 (Often). The Cronbach’s alphas of the scales were .92 for intrusive, and .86 for deliberate rumination.

Self-blame was measured with the Brief COPE Inventory (BCI; Carver, 1997). This inventory consists of 28 items corresponding to 14 coping strategies (two items each). For this study, we used only self-blame items (e.g., “I’ve been blaming myself for things that happened”). Participants rated each item on a 4-point Likert-type scale ranging from 1 (I have not been doing this at all) to 4 (I’ve been doing this a lot).

Single item questions measured additional variables such as age, education, relationship status, relationship status with the perpetrator (s), the time since the last violence incident, and received psychological help.

### Statistical procedures

Participants’ demographic data were summarized using descriptive statistics. Cronbach’s alpha was used to report the reliability of the scales. The relationship between variables was tested using Pearson correlations. r values around .10 are considered small, .30 medium, and .50 or higher large (Cohen, 1988). All significance tests were two-sided with a 5% nominal level of significance. These analyses were conducted using SPSS v. 26 software package.

Path analysis was used to examine the pathways from the centrality of event to PTG. This technique allows a series of structural regression equations to be analyzed simultaneously while evaluating how well the overall model fits the data. We developed a general model to test the proposed theoretical model described by Tedeschi et al. (2018). The path analysis used centrality of event, deliberate and intrusive rumination as independent variables, self-blame as a mediator between intrusive and deliberate rumination, and PTG as a dependent variable. Also, deliberate and intrusive rumination were used as mediators for the path from the centrality of event to PTG. It is known from our previous studies (e.g., Bakaitytė et al., 2021) that time after the event is related to PTG in IPV survivors, and intrusive rumination is usually expected to decrease with time (Tedeschi et al., 2018). For this reason, we also controlled for time since the last violence incident in PTG and intrusive rumination. We analyzed the model using Mplus statistical software (Version 8.5, Muthén and Muthén, 2017).

According to Hayes and Scharkow (2013), bias-corrected confidence intervals were used to provide more accurate weightings between Type I and Type II errors and a more precise assessment of indirect effects. Consequently, 5,000 bootstrap samples and 95% bias-corrected confidence intervals (CI) were used to determine the significance of indirect effects. An indirect effect is deemed statistically significant if the value of 0 is not included in the bias-corrected CI. The goodness of fit of the path models was assessed by examining the root mean squared error of approximation (RMSEA) and the standardized root mean squared residual (SRMR) (close to or smaller than .08), the comparative fit index (CFI) (close to or larger than .90), and the Tucker–Lewis index (TLI) (close to or larger than .90). These analyses were conducted using Mplus Version 8.2 (Muthén and Muthén, 2017).

As some items had missing values, we conducted a normed χ2 (χ2/df ratio) test to determine whether the data were missing at random. According to Bollen (1989), a value less than 2.0 indicates that data is missing at random and that the maximum likelihood techniques are appropriate for use. The normed χ2 value in this study was 1.49. Using full information maximum likelihood (FIML, Full Information Maximum Likelihood default in Mplus), analyses were conducted using all available data from the total sample (*N* = 200).

## Results

### Preliminary analysis

Correlation analysis (Table 2) revealed that PTG was positively related to centrality of event and deliberate rumination, and not related to intrusive rumination and self-blame. Centrality of event was positively related to all study variables. Intrusive rumination was positively correlated with deliberate rumination, and both types of rumination were positively correlated with self-blame.

**Table 2:** Correlations among study variables, and descriptive statistics.

	1	2	3	4	5
1. Posttraumatic growth	–				
2. Centrality of event	.44**	–			
3. Intrusive rumination	.09	.35**	–		
4. Deliberate rumination	.26**	.41**	.50**	–	
5. Self-blame	.09	.21**	.31**	.37**	–
*M*	2.88	3.22	1.24	1.19	1.91
*SD*	1.35	.85	.74	.74	.78

**p < .01.

### Path analysis

The constructed model fit the data well χ2 (390) = 736.071, *p* < .001, CFI = .920, TLI = .911, RMSEA = .067 [.059, .074], and SRMR =.063. All possible paths were added to the analysis, and time since the last violence incident was added as a control variable for intrusive rumination and PTG.

**Figure 1:**
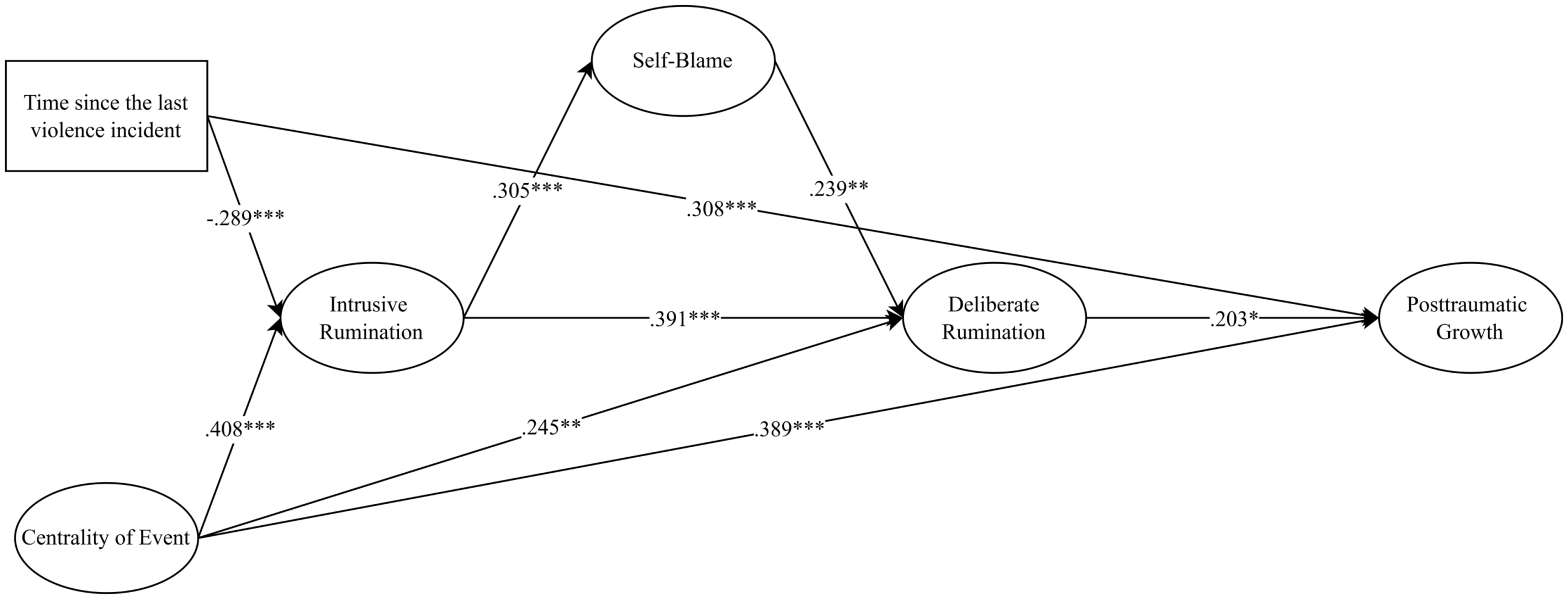
Structural equation model predicting post-traumatic growth. Only significant paths are indicated. Statistics are standardized regression coefficients. *p < 0.05, **p < 0.01, ***p < 0.001.

Results indicated that centrality of event was directly related to intrusive rumination, deliberate rumination, and PTG (Figure 1), showing that the more central IPV experience was to women, the more PTG, intrusive and deliberate rumination they experienced. The sequential indirect effect from centrality of event to PTG through intrusive and deliberate rumination was also significant (*B* = .032, 95% CI [.009, .071]), indicating that the relation between centrality of event and PTG is also related to higher levels of intrusive and deliberate rumination. Intrusive rumination was associated with deliberate rumination both directly and indirectly *via* self-blame (*B* = .073, 95% CI [.006, .213]), indicating that higher levels of intrusive rumination were associated with higher deliberate rumination, but this relation also goes through higher levels of self-blame. Deliberate rumination was positively associated with PTG, meaning that deliberate thinking about IPV experience led women to greater PTG. Time since the last violence incident was positively associated with PTG, and negatively with intrusive rumination, showing that the more time has passed from IPV experience, less intrusive rumination and more PTG women experience. The overall model explained almost 31% of PTG variance (R^2^ = .308, SE = .062, p < .001).

## Discussion

The current study aimed to test the theoretical pathway from the centrality of event to PTG including rumination (intrusive and deliberate) and self-blame in women victims and survivors of IPV. The overall results supported the main assumptions of the model of PTG (Tedeschi et al., 2018), indicating the path from centrality of event to PTG directly and indirectly through intrusive and deliberate rumination, and self-blame. However, found indirect effects raised some considerations, which are discussed in detail below.

In the current study, centrality of event was directly related to PTG, and this relation goes in line with studies not only with IPV (Bakaitytė et al., 2022) but also with other traumatic event survivors (Boals et al., 2010; Groleau et al., 2013; Lancaster et al., 2013). These results confirm one of the fundamental assumptions that “growth occurs when trauma assumes a central place in the life story” (Tedeschi and Calhoun, 1995, p. 85). As expected, centrality of event was also positively related to intrusive rumination, indicating that the more central the IPV experience becomes, the more women engaged in this type of rumination. This relation also supports theoretical assumptions indicating that traumatic experiences that are central to a person’s identity can initiate cognitive processing of trauma which starts from intrusive rumination (Tedeschi et al., 2018). Interestingly, centrality of event was also directly associated with deliberate rumination. Brooks et al. (2017), although investigated different directions (intrusive to deliberate rumination through centrality of event), also found the same positive association. These results indicate that centrality of event is the crucial factor affecting not only the beginning of the process of PTG but all major factors in it.

The sequential indirect effect was small yet significant, indicating that intrusive and deliberate rumination also work as mediators in the relation between centrality of event and PTG. This shows that the more women perceived their IPV experience as central the more intrusive rumination they experienced. Consequently, higher intrusive rumination was associated with higher deliberate rumination leading to more PTG. Similar results were found by Kramer et al. (2020) who instead of intrusive rumination investigated PTSD. The indirect path from centrality of event to PTG gives support for assumptions that intrusive thinking about IPV experience, although not related to PTG directly, transitions to more deliberate rumination in this way leading to PTG, as described in the model of PTG. However, the small indirect effect indicates the possibility that other factors noted in the model of PTG are involved in these relations, such as social support, disclosure, self-analysis (Tedeschi et al., 2018).

We found that self-blame indirectly affects the relation between intrusive and deliberate rumination, indicating that the transition from intrusive to deliberate rumination partly goes through self-blame. Self-blame is common among IPV survivors (Karakurt et al., 2014; Pereira et al., 2020) and originates in society which attributes the blame to victims rather than perpetrators (Kennedy and Prock, 2018). Ulloa et al. (2016) argue that, in cases of interpersonal violence, self-blame also reflects some control attributed to oneself. In this sense, self-blame works as a coping mechanism letting women sustain beliefs that they have control over what happens to them, which is very important in the context of IPV. In a violent relationship, the perpetrator puts his efforts to control the victim through violent and controlling behaviors, making the victim feel powerless and helpless (Filson et al., 2010). Thus, regaining control is an important task for survivors. Considering this, it can be assumed that self-blame contributes to regaining control and fosters more deliberate rumination eventually leading to PTG. However, as we did not measure actual perceived control, this assumption should be tested in future studies.

The positive effect of self-blame in the relation between intrusive and deliberate rumination could also indicate illusionary aspects of the PTG process. According to Zoellner and Maercker (2006), the link between PTG and coping efforts oriented toward avoidance rather than acceptance of reality represents an illusory side of PTG. The argument is that PTG has two sides - constructive, self-transcending, and self-deceptive, illusory (Maercker and Zoellner, 2004; Zoellner and Maercker, 2006). The PTG described by Tedeschi and Calhoun reflects the constructive side - struggle with traumatic experiences leads to personal transformation and positive changes. The illusory side reflects efforts to calm down by convincing oneself that something good came out of suffering. Authors emphasize that in the short run illusory efforts could indicate self-enhancing cognitions that help to reduce stress and if accompanied by deliberate thinking could eventually lead to actual growth (Maercker and Zoellner, 2004). However, the results of our study indicate the positive associations between self-blame and intrusive and deliberate rumination but not between self-blame and PTG which makes arguments about illusory PTG less plausible in the current context.

In this study, we asked participants to indicate what they have been doing to cope with the IPV experience, and we cannot distinguish if self-blame was a long-lasting, continuing coping strategy or a strategy that was used for some time and then changed to another. Considering this and the arguments of Zoellner and Mearcker, it is possible that women who blame themselves because of their IPV experience report PTG because they want to believe that, however horrible, this experience had some meaning and they gained something positive out of it, which is indicative of the illusory side of PTG. However, it is also possible that self-blame was part of the journey of coping with the IPV experience that involved many other factors which eventually helped them to achieve real positive changes. Based on the available data, it is not possible to indicate which explanation is true.

In conclusion, the current study confirmed some of the theoretical assumptions of the process of PTG and revealed problematic areas of its investigation. Results indicated that centrality of event is an important factor not only directly associated with PTG but also indirectly *via* intrusive and deliberate rumination. This indirect effect gave support to the argument that cognitive processing of IPV experience starting from intrusive rumination transitions to more deliberate rumination eventually leading to PTG. The investigation of this transition through the coping strategy of self-blame pointed out widely discussed debates about the real and illusory sides of PTG. However, this study shed some light on how coping should be investigated in the future to make more precise conclusions. Overall, the results of this study highlight the importance of a further and more in-depth examination of the process of PTG.

### Limitations and future directions

This study has some strengths and limitations. First, this is a cross-sectional study, and no causal assumptions could be made. Also, although Lithuania is currently increasingly WEIRD (western, educated, industrialized, rich, and democratic), the results of this study to some extent might be specific to the Northern European context. Moreover, we did not asked participants about their ethnicity as 5/6 of Lithuania’s population account for ethnic Lithuanians (Statistics Lithuania, 2020) and there is no accepted practice to ask participants about their ethnicity if research questions are not related to that. However, future studies should consider including an ethnic background as a possibly important factor associated with PTG in victims and survivors of IPV. Another limitation is that the current study asked participants to indicate what they have been doing to cope with IPV experience, and the responses do not represent their current coping strategies. It is possible that women blamed themselves to cope at first, but later they used different strategies, or some other factors influenced their self-blame, so results involving self-blame should be viewed with caution. Future studies investigating PTG of victims and survivors of IPV should include more IPV-related factors in the analysis. Such factors include current relationship status with the perpetrator, stalking, or continuing psychological abuse after leaving the abuser (especially when having children together) might be the factors that affect not only recovery processes but also the process of PTG. Moreover, the general practice in PTG research to use coping categories that include different strategies might not be as informative and even misleading (Bonanno and Burton, 2013), for this reason, the investigation of separate coping strategies in the process of PTG might be more useful. Considering the dynamic nature of coping strategies, it is important to investigate these strategies longitudinally to see how they change and how this affects PTG. This could give more answers about illusory and real positive changes. Also, studies have shown that other factors, such as personality traits (Shakespeare-Finch et al., 2005), emotion regulation skills (Larsen and Berenbaum, 2015), or perceived control (Frazier et al., 2004) are related to PTG experience and coping strategies, especially self-blame. Thus, inclusion of these factors in future studies may provide more insight into what interventions might be appropriate for different women who have experienced IPV. Finally, the assumptions about illusionary PTG indicate that cognitive processing in the PTG process is complex, involving many different environmental and intrapersonal factors that need further investigation including more in-depth qualitative and longitudinal studies.

The strengths of the current study involve a relatively big sample of victims and survivors of IPV that is difficult to recruit. Also, this is one of the few studies investigating the cognitive processing part of the model of PTG as a whole, and to our knowledge, this is the first study investigating the path to PTG in IPV survivors. The results of this study highlight the importance to consider factors specific to the traumatic context while investigating PTG and draw attention to the complexity of the process of PTG that need further investigation.

### Final remarks

The current study involves self-blame as a coping mechanism of women survivors of IPV and the results including this strategy can give the false impression that self-blame leading to positive changes after experiencing IPV is a positive thing. As indicated in the discussion, self-blame as a coping strategy in a way protects victims’ belief system for a short time but in no way it is a positive or desirable path to PTG. The origins of self-blame lie in society’s tendency to stigmatize and blame the victim rather than the abuser and current results represent this sad reality that IPV survivors not only have to undergo the consequences of IPV but also must endure feelings of self-blame which society is the culprit. Therefore, with the results of our study, we are in no way attributing self-blame as a positive factor. On the contrary, we are informing that it is there, affecting these women, and there are a lot of questions that need to be answered.

## Data availability statement

The raw data supporting the conclusions of this article will be made available by the authors, without undue reservation.

## Ethics statement

The studies involving human participants were reviewed and approved by the Committee of Psychological Research Ethics, Institute of Psychology, Mykolas Romeris University. The participants provided their written informed consent to participate in this study.

## Author contributions

AB and RŽ prepared materials for data collection. AB conceptualized the study and wrote the first draft of the manuscript. AB and AP-M designed the study and conducted the statistical analysis. All authors reviewed the manuscript, made revisions, and approved the submitted version.

## Funding

This work was supported by a grant from the Research Council of Lithuania, [P-MIP-17-132] and is part of the I+D+i project PID2020-116658GB-I00PSI, financed by MCIN/ AEI/10.13039/501100011033/.

## Conflict of interest

The authors declare that the research was conducted in the absence of any commercial or financial relationships that could be construed as a potential conflict of interest.

## Publisher’s note

All claims expressed in this article are solely those of the authors and do not necessarily represent those of their affiliated organizations, or those of the publisher, the editors and the reviewers. Any product that may be evaluated in this article, or claim that may be made by its manufacturer, is not guaranteed or endorsed by the publisher.

## References

[ref1] Ansara D. L. Hindin M. J. (2011). Psychosocial consequences of intimate partner violence for women and men in Canada. J. Interpers. Viol. 26, 1628–1645. doi: 10.1177/0886260510370600, PMID: 20501897

[ref2] Bakaitytė A. Kaniušonytė G. Truskauskaitė-Kunevičienė I. Žukauskienė R. (2022). Longitudinal investigation of posttraumatic growth in female survivors of intimate partner violence: the role of event centrality and identity exploration. J. Interpers. Viol. 37:864. doi:10.1177/0886260520920864 32410496

[ref3] Bakaitytė A. Kaniušonytė G. Žukauskienė R. (2021). Posttraumatic growth, centrality of event, trauma symptoms and resilience: profiles of women survivors of intimate partner violence. J. Interpers. Viol. 37, NP20168–NP20189. doi: 10.1177/08862605211050110, PMID: 34658266 PMC9554379

[ref4] Berntsen D. Rubin D. C. (2006). The centrality of event scale: a measure of integrating a trauma into one’s identity and its relation to post-traumatic stress disorder symptoms. Behav. Res. Ther. 44, 219–231. doi: 10.1016/j.brat.2005.01.009, PMID: 16389062 PMC3974102

[ref5] Boals A. Steward J. M. Schuettler D. (2010). Advancing our understanding of posttraumatic growth by considering event centrality. J. Loss Trauma 15, 518–533. doi: 10.1080/15325024.2010.519271

[ref6] Bollen K. A. (1989). Structural Equations With Latent Variables. New York: John Wiley & Sons.

[ref7] Bonanno G. A. Burton C. L. (2013). Regulatory flexibility: an individual differences perspective on coping and emotion regulation. Perspect. Psychol. Sci. 8, 591–612. doi: 10.1177/1745691613504116 26173226

[ref8] Brooks M. Graham-Kevan N. Lowe M. Robinson S. (2017). Rumination, event centrality, and perceived control as predictors of post-traumatic growth and distress: the cognitive growth and stress model. Br. J. Clin. Psychol. 56, 286–302. doi: 10.1111/bjc.12138, PMID: 28464228

[ref9] Brooks M. Graham-Kevan N. Robinson S. Lowe M. (2019). Trauma characteristics and posttraumatic growth: the mediating role of avoidance coping, intrusive thoughts, and social support. Psychol. Trauma Theory Res. Pract. Policy 11, 232–238. doi: 10.1037/tra0000372, PMID: 29723030

[ref10] Cann A. Calhoun L. G. Tedeschi R. G. Taku K. Vishnevsky T. Triplett K. N. (2010). A short form of the posttraumatic growth inventory. Anxiety Stress Coping 23, 127–137. doi: 10.1080/10615800903094273, PMID: 19582640

[ref11] Cann A. Calhoun L. G. Tedeschi R. G. Triplett K. N. Vishnevsky T. Lindstrom C. M. (2011). Assessing posttraumatic cognitive processes: the event related rumination inventory. Anxiety Stress Coping 24, 137–156. doi: 10.1080/10615806.2010.529901, PMID: 21082446

[ref12] Carver C. S. (1997). You want to measure coping but your protocol’s too long: consider the brief COPE. Int. J. Behav. Med. 4, 92–100. doi: 10.1207/s15327558ijbm0401_6, PMID: 16250744

[ref13] Chandan J. Thomas T. Bradbury-Jones C. Russell R. Bandyopadhyay S. Nirantharakumar K. (2020). Female survivors of intimate partner violence and risk of depression, anxiety and serious mental illness. Br. J. Psychiatry 217, 562–567. doi: 10.1192/bjp.2019.124, PMID: 31171045

[ref14] Chi D. de Terte I. Gardner D. (2022). Posttraumatic growth and posttraumatic stress symptoms in people with HIV. AIDS Behav. 26, 3688–3699. doi: 10.1007/s10461-022-03697-3, PMID: 35666361 PMC9550787

[ref15] Cobb A. R. Tedeschi R. G. Calhoun L. G. Cann A. (2006). Correlates of posttraumatic growth in survivors of intimate partner violence. J. Trauma. Stress. 19, 895–903. doi: 10.1002/jts.20171, PMID: 17195973

[ref16] Doane N. J. K. (2010). Predictors of Posttraumatic Growth, Shame, and Posttraumatic Stress Symptoms in Survivors of Intimate Partner Violence: The Roles of Social Support and Coping. [Doctoral Dissertation, The University of Montana], Graduate Student Theses, Dissertations, & Professional Papers. *Vol. 765.* Retrieved from http://scholarworks.umt.edu/etd/765.

[ref500] Cohen J. (1988). Statistical power analysis for the behavioral sciences. 2nd Edn. Lawrence Erlbaum Associates., PMID: 512837

[ref17] Elderton A. Berry A. Chan C. (2017). A systematic review of posttraumatic growth in survivors of interpersonal violence in adulthood. Trauma Viol. Abuse 18, 223–236. doi: 10.1177/1524838015611672, PMID: 26459504

[ref18] Filson J. Ulloa E. Runfola C. Hokoda A. (2010). Does powerlessness explain the relationship between intimate partner violence and depression? J. Interpers. Viol. 25, 400–415. doi: 10.1177/0886260509334401, PMID: 19487687

[ref19] Frazier P. Tashiro T. Berman M. Steger M. Long J. (2004). Correlates of levels and patterns of positive life changes following sexual assault. J. Consult. Clin. Psychol. 72, 19–30. doi: 10.1037/0022-006X.72.1.19, PMID: 14756611

[ref20] Freedle A. Kashubeck-West S. (2021). Core belief challenge, rumination, and posttraumatic growth in women following pregnancy loss. Psychol. Trauma Theory Res. Pract. Policy 13, 157–164. doi: 10.1037/tra0000952, PMID: 32881569

[ref21] Groleau J. M. Calhoun L. G. Cann A. Tedeschi R. G. (2013). The role of centrality of events in posttraumatic distress and posttraumatic growth. Psychol. Trauma Theory Res. Pract. Policy 5, 477–483. doi: 10.1037/a0028809

[ref22] Hayes A. F. Scharkow M. (2013). The relative trustworthiness of inferential tests of the indirect effect in statistical mediation analysis: does method really matter? Psychol. Sci. 24, 1918–1927. doi: 10.1177/0956797613480187, PMID: 23955356

[ref23] Janoff-Bulman R. (1979). Characterological versus behavioral self-blame: inquiries into depression and rape. J. Pers. Soc. Psychol. 37, 1798–1809. doi: 10.1037/0022-3514.37.10.1798, PMID: 512837

[ref24] Jia X. Liu X. Ying L. Lin C. (2017). Longitudinal relationships between social support and posttraumatic growth among adolescent survivors of the Wenchuan earthquake. Front. Psychol. 8:1275. doi: 10.3389/fpsyg.2017.01275, PMID: 28804469 PMC5532511

[ref25] Joseph S. Linley P. A. (2005). Positive adjustment to threatening events: an organismic valuing theory of growth through adversity. Rev. Gen. Psychol. 9, 262–280. doi: 10.1037/1089-2680.9.3.262

[ref26] Kamijo N. Yukawa S. (2018). The role of rumination and negative affect in meaning making following stressful experiences in a Japanese sample. Front. Psychol. 9:2404. doi: 10.3389/fpsyg.2018.02404, PMID: 30546340 PMC6279863

[ref27] Karakurt G. Smith D. Whiting J. (2014). Impact of intimate partner violence on women’s mental health. J. Fam. Viol. 29, 693–702. doi: 10.1007/s10896-014-9633-2, PMID: 25313269 PMC4193378

[ref28] Kennedy A. C. Prock K. A. (2018). “I still feel like I am not Normal”: a review of the role of stigma and stigmatization among female survivors of child sexual abuse, sexual assault, and intimate partner violence. Trauma Viol. Abuse 19, 512–527. doi: 10.1177/1524838016673601, PMID: 27803311

[ref29] Kramer L. B. Whiteman S. E. Witte T. K. Silverstein M. W. Weathers F. W. (2020). From trauma to growth: the roles of event centrality, posttraumatic stress symptoms, and deliberate rumination. Traumatology 26, 152–159. doi: 10.1037/trm0000214

[ref30] Kunz S. Joseph S. Geyh S. Peter C. (2018). Coping and posttraumatic growth: a longitudinal comparison of two alternative views. Rehabil. Psychol. 63, 240–249. doi: 10.1037/rep0000205, PMID: 29878829

[ref31] Lafarge C. Usher L. Mitchell K. Fox P. (2020). The role of rumination in adjusting to termination of pregnancy for fetal abnormality: rumination as a predictor and mediator of posttraumatic growth. Psychol. Trauma Theory Res. Pract. Policy 12, 101–109. doi: 10.1037/tra0000440, PMID: 30816771

[ref32] Lancaster S. L. Kloep M. Rodriguez B. F. Weston R. (2013). Event centrality, posttraumatic cognitions, and the experience of posttraumatic growth. J. Aggress. Maltreat. Trauma 22, 379–393. doi: 10.1080/10926771.2013.775983

[ref33] Larsen S. E. Berenbaum H. (2015). Are specific emotion regulation strategies differentially associated with posttraumatic growth versus stress? J. Aggress. Maltreat. Trauma 24, 794–808. doi: 10.1080/10926771.2015.1062451

[ref34] Lazarus R. S. Folkman S. (1984). Stress, appraisal, and coping. New York: Springer.

[ref35] Lee D. Yu E. S. Kim N. H. (2020). Resilience as a mediator in the relationship between posttraumatic stress and posttraumatic growth among adult accident or crime victims: the moderated mediating effect of childhood trauma. Eur. J. Psychotraumatol. 11:1704563. doi: 10.1080/20008198.2019.1704563, PMID: 32002138 PMC6968590

[ref36] Lilly M. M. Howell K. H. Graham-Bermann S. (2015). World assumptions, religiosity, and PTSD in survivors of intimate partner violence. Viol. Again. Women 21, 87–104. doi: 10.1177/1077801214564139, PMID: 25540252

[ref37] London M. J. Mercer M. C. Lilly M. M. (2020). Considering the impact of early trauma on coping and pathology to predict posttraumatic growth among 9-1-1 Telecommunicators. J. Interpers. Viol. 35, 4709–4731. doi: 10.1177/0886260517716942, PMID: 29294814

[ref38] Lowe S. R. James P. Arcaya M. C. Vale M. D. Rhodes J. E. Rich-Edwards J. (2020). Do levels of posttraumatic growth vary by type of traumatic event experienced? An analysis of the nurses’ health study II. Psychol. Trauma Theory Res. Pract. Policy 14, 1221–1229. doi: 10.1037/tra0000554, PMID: 32212776 PMC7529660

[ref39] Maercker A. Zoellner T. (2004). The Janus face of self-perceived growth: toward a two-component model of posttraumatic growth. Psychol. Inq. 15, 41–48. http://www.jstor.org/stable/20447200

[ref40] Maguen S. Vogt D. S. King L. A. King D. W. Litz B. T. Knight S. J. (2011). The impact of killing on mental health symptoms in gulf war veterans. Psychol. Trauma Theory Res. Pract. Policy 3, 21–26. doi: 10.1037/a0019897

[ref41] Matheson F. I. Daoud N. Hamilton-Wright S. Borenstein H. Pedersen C. O’Campo P. (2015). Where did she go? The transformation of self-esteem, self-identity, and mental well-being among women who have experienced intimate partner violence. Womens Health Iss. 25, 561–569. doi: 10.1016/j.whi.2015.04.006, PMID: 26116987

[ref42] Meyerson D. A. Grant K. E. Carter J. S. Kilmer R. P. (2011). Posttraumatic growth among children and adolescents: a systematic review. Clin. Psychol. Rev. 31, 949–964. doi: 10.1016/j.cpr.2011.06.003, PMID: 21718663

[ref43] Morgan J. K. Desmarais S. L. (2017). Associations between time since event and posttraumatic growth among military veterans. Mil. Psychol. 29, 456–463. doi: 10.1037/mil0000170

[ref44] Muthén L. K. Muthén B. (2017). Mplus User’s Guide. Eighth Edn. Los Angeles: Muthén & Muthén.

[ref45] Nishi D. Matsuoka Y. Kim Y. (2010). Posttraumatic growth, posttraumatic stress disorder and resilience of motor vehicle accident survivors. Biopsychosoc. Med. 4, 1–6. doi: 10.1186/1751-0759-4-7, PMID: 20573276 PMC2914073

[ref46] Ogińska-Bulik N. (2016). The role of rumination in the occurrence of positive effects of experienced traumatic events. Health Psychol. Rep. 4, 321–331. doi: 10.5114/hpr.2016.60915

[ref47] Pereira M. E. Azeredo A. Moreira D. Brandão I. Almeida F. (2020). Personality characteristics of victims of intimate partner violence: a systematic review. Aggress. Viol. Behav. 52:101423. doi: 10.1016/j.avb.2020.101423

[ref48] Platte S. Wiesmann U. Tedeschi R. G. Kehl D. (2022). Coping and rumination as predictors of posttraumatic growth and depreciation. Chin. J. Traumatol. 25, 264–271. doi: 10.1016/j.cjtee.2022.02.001, PMID: 35304016 PMC9458987

[ref49] Prati G. Pietrantoni L. (2009). Optimism, social support, and coping strategies as factors contributing to posttraumatic growth: a meta-analysis. J. Loss Trauma 14, 364–388. doi: 10.1080/15325020902724271

[ref50] Reich C. M. Jones J. M. Woodward M. J. Blackwell N. Lindsey L. D. Beck J. G. (2015). Does self-blame moderate psychological adjustment following intimate partner violence? J. Interpers. Viol. 30, 1493–1510. doi: 10.1177/0886260514540800, PMID: 24997098

[ref51] Shakespeare-Finch J. Gow K. Smith S. (2005). Personality, coping and posttraumatic growth in emergency ambulance personnel. Traumatology 11, 325–334. doi: 10.1177/1534765605011004

[ref52] Statistics Lithuania (2020). Residents of Lithuania. Statistics Lithuania. Available at: http://osp. stat.gov.lt/lietuvosgyventojai-2020/salies-gyventojai/gyventoju-skaicius-ir-sudetis

[ref53] Stockton H. Hunt N. Joseph S. (2011). Cognitive processing, rumination, and posttraumatic growth. J. Trauma. Stress. 24, 85–92. doi: 10.1002/jts.20606 21268118

[ref54] Tedeschi R. G. Calhoun L. G. (1995). Trauma and Transformation: Growing in the Aftermath of Suffering. Sage Publication, Inc. doi: 10.4135/9781483326931

[ref55] Tedeschi R. G. Shakespeare-Finch J. Taku K. Calhoun L. G. (2018). Posttraumatic growth: Theory, research, and applications. New York: Routledge doi: 10.4324/9781315527451

[ref56] Ulloa E. Guzman M. L. Salazar M. Cala C. (2016). Posttraumatic growth and sexual violence: a literature review. J. Aggress. Maltreat. Trauma 25, 286–304. doi: 10.1080/10926771.2015.1079286, PMID: 29503522 PMC5831550

[ref57] Valdez C. E. Lilly M. M. (2015). Posttraumatic growth in survivors of intimate partner violence: an assumptive world process. J. Interpers. Violence 30, 215–231. doi: 10.1177/0886260514533154, PMID: 24850765

[ref58] World Health Organization and Pan American Health Organization (2012). Understanding and Addressing Violence Against Women: Intimate Partner Violence. World Health Organization. Available at: https://apps.who.int/iris/handle/10665/77432

[ref60] Young M. D. (2007). Finding Meaning in the Aftermath of Trauma: Resilience and Posttraumatic Growth in Female Survivors of Intimate Partner Violence (Publication No. 3258728) [Doctoral Dissertation, The University of Montana].ProQuest Dissertations and Theses Globa. Retrieved from http://www.proquest.com/openview/1e47887d65fd9970b9291ec11c9e030e/1?pq-origsite=gscholar

[ref61] Zoellner T. Maercker A. (2006). Posttraumatic growth in clinical psychology - a critical review and introduction of a two component model. Clin. Psychol. Rev. 26, 626–653. doi: 10.1016/j.cpr.2006.01.008, PMID: 16515831

